# Histone Modifications and the Maintenance of Telomere Integrity

**DOI:** 10.3390/cells8020199

**Published:** 2019-02-25

**Authors:** Meagan Jezek, Erin M. Green

**Affiliations:** Department of Biological Sciences, University of Maryland Baltimore County, Baltimore, MD 21250, USA; mjezek1@umbc.edu

**Keywords:** telomeres, histone modifications, post-translational modifications, telomere position effect, histone acetylation, histone methylation

## Abstract

Telomeres, the nucleoprotein structures at the ends of eukaryotic chromosomes, play an integral role in protecting linear DNA from degradation. Dysregulation of telomeres can result in genomic instability and has been implicated in increased rates of cellular senescence and many diseases, including cancer. The integrity of telomeres is maintained by a coordinated network of proteins and RNAs, such as the telomerase holoenzyme and protective proteins that prevent the recognition of the telomere ends as a DNA double-strand breaks. The structure of chromatin at telomeres and within adjacent subtelomeres has been implicated in telomere maintenance pathways in model systems and humans. Specific post-translational modifications of histones, including methylation, acetylation, and ubiquitination, have been shown to be necessary for maintaining a chromatin environment that promotes telomere integrity. Here we review the current knowledge regarding the role of histone modifications in maintaining telomeric and subtelomeric chromatin, discuss the implications of histone modification marks as they relate to human disease, and highlight key areas for future research.

## 1. Introduction

Due to the linear nature of eukaryotic chromosomes, their ends must be protected in order to prevent the progressive loss of sequence with each round of cell division, which can eventually lead to genomic instability, disease, and cell death [1,2]. Telomeres are the nucleoprotein structures found at the ends of linear chromosomes that serve as protective caps, prevent chromosomal ends from being treated as double-strand breaks, and counteract telomere shortening during replication [3,4]. Telomeres have a number of associated proteins that aid in this function, many of which act to maintain telomere length directly, such as telomerase, or protect the ends from recognition as DNA double-strand breaks, such as the shelterin complex in mammals and functionally orthologous proteins in model systems [3,4,5]. In addition to the proteins bound to chromosome ends, the maintenance of highly organized and well-regulated chromatin structure at or near telomeres has been linked to the preservation of telomere integrity. Histones, the protein component of nucleosomes, are frequently sites of post-translational modifications (PTM) such as acetylation, methylation, and ubiquitylation. Altered regulation of these PTMs can result in disrupted telomere dynamics, which have been linked to cell senescence and a number of diseases, including some cancers [6,7]. However, the molecular signaling between chromatin, telomeres, and associated protective proteins is not well understood. In this review, we will specifically focus on understanding the roles of histone PTMs within the telomeric and subtelomeric chromatin in the maintenance of telomere integrity, and discuss examples of chromatin modifications that have been implicated in protecting chromosome ends. The yeasts *Saccharomyces cerevisiae* and *Schizosacchromyces pombe* have long served as models for understanding telomere function. We focus on key findings described in yeast, and highlight findings connecting chromatin structure, telomere function, and disease in mammalian systems.

## 2. Histone Modifications and Chromatin Dynamics

The basic unit of chromatin, the nucleosome, consists of 146 base pairs of DNA wrapped around an octamer of histone proteins (H2A, H2B, H3, and H4). One of the main mechanisms for the regulation of chromatin structure is through the post-translational modification (PTM) of histones, which most commonly occur on the amino acid residues lysine, arginine, serine, tyrosine, and threonine. The addition or removal of these chemical groups to or from specific residues on histones can both alter histone–DNA association and serve as a signal to be recognized by chromatin-modifying or remodeling proteins, which ultimately alter transcriptional activity. Post-translational histone modifications can include phosphorylation, methylation, acetylation, sumoylation, and ubiquitylation. Crosstalk between these different modifications results in context-dependent mechanisms through which gene expression and other genomic functions are regulated [8,9,10,11,12].

The enzymes that facilitate the addition or removal of these marks have been termed “writers” and “erasers,” respectively. Examples of writers include histone acetyltransferases (HATs), arginine and lysine methyltransferases (PRMTs and KMTs, respectively), ubiquitin ligases, and kinases. Conversely, the respective erasers include histone deacetylases (HDACs), arginine and lysine demethylases (PRDMs and KDMs, respectively), deubiquitinases, and phosphatases. Proteins that recognize and bind to PTMs, or to an unmodified region of a histone, are classified as “readers”. These proteins typically rely on specialized domains, a few of which include the methyl–lysine or acetyl–lysine chromodomains, PHD fingers, or bromodomains [13,14,15]. The functional outcome of a particular modification therefore depends on the reader protein that recognizes it within a specific biological context. The overall effect of the interaction of these proteins with chromatin is the dynamic regulation of factors that interact with DNA for critical processes, such as transcription, replication, and repair, which are essential to the maintenance of a healthy cell physiology.

### 2.1. Chromatin Dynamics at Telomeres

**Figure 1:**
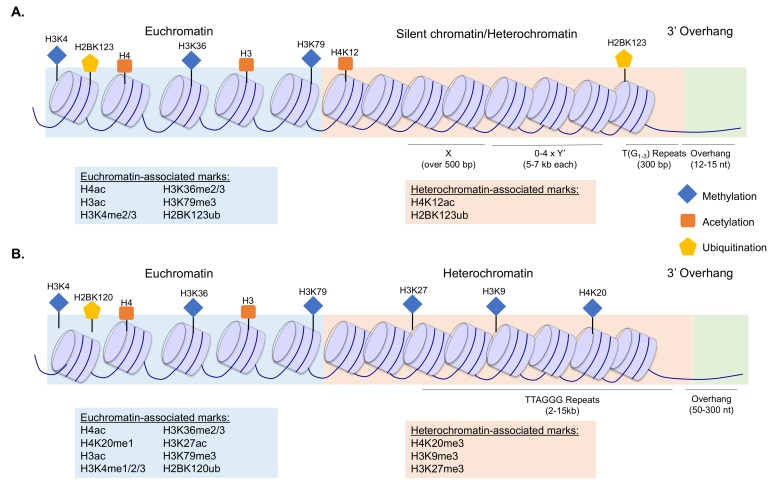
Schematic of budding yeast (**A**) and human (**B**) telomeres and subtelomeres, indicating the primary sequence elements, the general chromatin structure, and the distribution of histone modifications within these regions. Euchromatin-associated and heterochromatin-associated marks are indicated in the figure as shown in the key, and further specified in the text boxes. To improve visualization of the chromatin structure, the telomeres are not drawn as t-loops here. References describing localization patterns of the histone modifications are provided within the main text.

A requirement for proper telomere function is the maintenance of the adjacent chromatin landscape. Disruptions to chromatin structure through the mutation or altered expression of chromatin-modifying factors have been linked to telomere dysfunction and genomic instability [6,16,17,18]. Budding yeast telomeres consist of T-(G_1–3_) repeats with 3’ overhangs of the G-rich strand, and the adjacent subtelomeres contain repeat sequences known as X elements, which are found on all chromosome ends, and Y’ elements, which are found in variable numbers in a subset of subtelomeres [4] (Figure 1A). In humans, telomeres consist of an average of 2–15 kb of repeats of the sequence TTAGGG and have a 3’ overhang of the G-rich strand (Figure 1B), which is thought to fold back into the double-stranded DNA, forming a structure known as the t-loop [19]. The t-loop, along with the multisubunit protein complex shelterin, which binds telomeric repeats, acts to protect chromosome ends and prevent inappropriate signaling to the DNA damage response machinery [20].

In yeast, the telomeric repeats do not appear to be associated with nucleosomes; however, subtelomeric DNA is associated with nucleosomes and is thought to be predominantly composed of transcriptionally silent chromatin [21] (Figure 1A). The subtelomeres are adjacent to more transcriptionally active chromatin and this heterochromatin–euchromatin boundary is maintained by a highly regulated set of chromatin modifiers and remodelers and a distinct histone PTM distribution [8]. The loss of silencing factors and disruptions to the repressed chromatin within subtelomeres have been linked to telomere dysfunction and phenotypes such as aberrant transcription, chromosome fusions, telomere shortening, and cellular senescence [6,7,22].

The nature of chromatin at mammalian telomeres is subject to more debate. While there is significant evidence indicating that telomeres are likely to be largely heterochromatic, the existence of heterochromatin at telomeres may vary in different cell types or under different conditions [23], and there are methodological challenges to clearly defining the composition of telomeric chromatin [24]. Nonetheless, as described below, transcriptionally repressed chromatin and altered patterns of histone acetylation are linked to defects in telomere maintenance, indicating that a heterochromatic environment likely contributes to telomere function. Additionally, a recent report targeted the heterochromatin protein HP1α to a specific telomere and observed attenuation of telomerase activity and protection from damage upon increased heterochromatinization [25], providing evidence that telomere homeostasis is promoted by local heterochromatin.

In diverse systems, genes adjacent to telomeric regions are often lowly expressed and subject to transcriptional silencing, an observation known as the telomere position effect, or TPE [26]. TPE is thought to arise from the high local concentration of silencing factors and repressive chromatin proteins. Despite a generally repressive environment, transcription does occur at telomeres, primarily in the form of the noncoding telomere repeat containing RNA, or TERRA, which is transcribed from the C-rich strand [27,28]. The degree of silencing of sub-telomeric coding genes is also variable between telomeres and amongst different organisms. In yeast, TPE silences genes within subtelomeres, and the degree of TPE depends on telomere length, subnuclear localization, the presence and combination of X and Y’ elements, and the concentration and localization of repressive factors, such as histone modifiers [26,29,30]. In mammalian cells, TPE was initially described using reporter genes inserted adjacent to telomeres [31,32,33]. However, more recent work has shown that TPE can affect genes at a much greater distance from heterochromatic subtelomeres. This phenomenon, known as telomere position effect over long distances, or TPE-OLD, is due to the location of the telomeres in three-dimensional space relative to otherwise transcriptionally active regions [34,35]. In addition to heterochromatic silencing factors spreading into euchromatin, when mammalian telomeres form t-loops, silencing factors affect genes more distal from the telomere ends [36]. This mechanism has been shown to influence a number of genes [35,37], including the *hTERT* gene in humans, which encodes the telomerase reverse transcriptase [35,38]. When telomeres are long, their looping conformation leads to the repression of *hTERT* transcription. However, as telomeres shorten, the silencing loop structure is no longer in close proximity to *hTERT*, leading to the transcriptional activation of *hTERT*, and therefore to increased telomerase activity, which counteracts the telomere shortening. These findings suggest that TPE may function to both regulate gene expression and maintain telomere length as cells senesce.

#### 2.1.1. Histone Acetylation at Telomeres

Acetylation of lysines on histones lessens their overall positive charge, weakening histone–DNA interactions and providing a permissible chromatin landscape for transcriptional activation. Silent regions of the genome, such as within yeast subtelomeres and at mammalian heterochromatin, often contain hypo-acetylated histones. Histone acetylation marks that are typically associated with transcriptionally active areas of the genome include those on H2AK7, H2BK11 and K16, H3 at lysines 9, 14, 18, 27, and 56, and on H4 at lysines 5, 8, 12, and 16 [39,40]. In yeast, a predominant feature of subtelomeres is the silent information regulator, or SIR, histone deacetylase complex, which is composed of Sir2, Sir3, and Sir4. This complex localizes to chromatin in a DNA sequence-independent manner via the interaction of Sir3 with the telomere-associated Ku complex and Rap1 protein [41,42,43,44]. The deacetylation of lysine 16 on histone H4 is performed by the catalytic component Sir2, an NAD^+^-dependent deacetylase [42]. The absence of the SIR complex leads to the de-repression of a relatively small subset of subtelomeric genes [45], suggesting that other factors are also key contributors to subtelomeric gene repression. Interestingly, the loss of Sir2 and the resulting increase in H4K16ac at subtelomeres is associated with an increase in the transcription of TERRA, which negatively regulates telomere length [46,47,48,49,50], linking SIR-dependent transcriptional control with telomere length homeostasis. In mammals, the Sir2 family member SIRT6 is an H3K9ac, H3K18ac, and H3K56ac deacetylase [51,52,53] that is associated with telomeres and is required for preventing telomere dysfunction and premature senescence [51,54]. SIRT6 has been shown to be required for the TPE of normally silent genes [55], indicating that mammalian telomeres also rely on chromatin regulation by deacetylation to maintain telomere integrity and the repression of telomere-adjacent genes.

While the majority of lysines on histones are hypoacetylated near telomeres, it has been shown in budding yeast that H4K12ac, established by the NuA4 acetyltransferase complex, is found in telomeric regions [56]. This mark appears to prevent inappropriate SIR complex binding and over-compaction of the subtelomeres in order to promote the accessibility of the chromatin to transcriptional regulators and telomere maintenance proteins. The loss of H4K12ac results in defects in telomere length and recombination mechanisms in the absence of telomerase [56], suggesting that acetylation within the largely repressive environment of yeast subtelomeres also plays a positive role in telomere maintenance pathways. In humans, hypoacetylation and repressive methylation marks have been identified within telomeric heterochromatin (Figure 1B), and euchromatic marks are associated with subtelomeres; however, there is relatively little understanding of the requirements for the appropriate balance of these marks at telomeric and subtelomeric regions, and the extent of the plasticity of human telomeric heterochromatin has not been fully explored.

#### 2.1.2. Histone Methylation at Telomeres

In contrast to histone acetylation, which is predominantly associated with gene activation, the addition of methyl groups to histone lysine [57] or arginine [58] residues is context-dependent, resulting in either activation or repression depending on the reader protein that binds a given methyl mark. Histone methyl marks known to play a role in repression at heterochromatic regions include H3K9me3, H3K27me3, H3K79me3, and H4K20me3 [16,24,59,60,61,62,63,64,65] (Figure 1B). While H3K9, H3K27, and H4K20 tri-methylation do not occur in budding yeast, these three modifications are repressive marks in mammalian systems and *S. pombe*. The proper distribution of histone methylation marks at telomeric and subtelomeric regions is established through multiple mechanisms, some of which are not completely understood; however, a recent study identified a link between the non-coding RNA TERRA and the establishment of heterochromatic marks at telomere repeats in human cell lines [66]. This work demonstrated that TERRAs originating from the human chromosome 20q bind to PRC2 and aid in the deposition of H3K27me3 to telomeric regions, followed by H3K9me3 and H4K20me3 deposition and HP1 recruitment. These observations not only suggest that TERRA is an important regulator of histone methylation and heterochromatin formation at telomeres, but also indicate the possibility of feedback mechanisms that link TERRA expression and heterochromatin maintenance at telomeres.

The H3K79 methyltransferase Dot1, or disruptor of telomeric silencing, was originally described for its role in promoting the silencing of subtelomeric genes in yeast and its loss leads to defects in telomere length [67]. It has been proposed that it binds to a region of the histone H4 tail in a mutually exclusive manner with Sir3, which is required for H4K16 deacetylation. The binding of Dot1 to the H4 tail in euchromatin is thought to prevent the binding of Sir3 to euchromatic regions, thereby maintaining its activity at subtelomeric heterochromatin [68,69,70]. However, there is debate regarding the precise role of Dot1 in subtelomere silencing regulation, as many of the initial experiments relied on the use of reporter genes which were later revealed to be indicative of nucleotide levels in the cell, rather than of subtelomere silencing per se [71,72]. There are indications that Dot1 is required for endogenous gene silencing at some telomeres [71], although it may play a more specialized role rather than a global role in TPE. In mouse ES cells, H3K79 methylation appears more enriched at subtelomeres than within the heterochromatic telomeres [62]. However, the loss of mouse *Dot1L* alters the distribution of marks within telomeres, including decreasing the repressive mark H4K20me3 and increasing the mark of euchromatin H3K9ac [62]. Additionally, a knockout of the Dot1L function results in elongated telomeres and the activation of the alternative lengthening of telomeres (ALT) pathway [62]. Together, these data suggest that Dot1L contributes to the maintenance of telomere heterochromatin, which may directly or indirectly impact telomere length. However, further investigation is required to specify the role of Dot1L in telomere maintenance, and to identify potential mechanisms by which it regulates chromatin structure and telomere function.

Gene silencing within subtelomeric regions and the regulation of telomere length are also known to depend on H3K4 methylation, particularly in yeast and likely in mammalian systems [73,74,75,76,77,78,79,80,81,82]. H3K4 tri-methylation is commonly found at the 5’ end of actively transcribed genes, whereas di- and mono-methylation are typically found within gene bodies and at 3’ ends, respectively [83,84,85]. In yeast, the enzyme which catalyzes H3K4me1, me2, and me3 is Set1/COMPASS, which is recruited to RNA pol II co-transcriptionally [86]. Set1 and H3K4 methylation have also been linked to the regulation of silent regions such as the rDNA and telomeres [76,81,87,88] and more general gene repression pathways including ribosomal protein genes [89] and meiotic differentiation genes [90,91]. The loss of Set1 is also known to result in a number of telomere-linked phenotypes, including defective silencing, altered telomere length, and the disrupted subnuclear localization of telomeres [81,82,92,93,94]. In mammalian cells, H3K4 methylation and the methyltransferase MLL1 were found to be associated with telomeres and to regulate TERRA expression in a p53-dependent manner upon the uncapping of telomeres [73], suggesting that H3K4 methylation may play a role in mammalian telomere maintenance as well, although the molecular details of its role have not been thoroughly investigated.

In budding yeast, a proposed explanation for defective telomere silencing in the absence of Set1 involves the titration of the SIR deacetylase complex away from the euchromatin–heterochromatin boundary, leading to increased histone acetylation [95,96]. However, we recently demonstrated that SIR protein localization and the associated H4K16ac mark are not dramatically altered in cells lacking Set1 [78], which generally supports the emerging model that multiple repressive mechanisms may exist at telomeres and that Set1 likely acts independently of SIR-mediated silencing [45,97]. Set1 has also been shown to have some roles independent of its methyltransferase activity [98,99,100,101], and there is some indication that its function at telomeres may rely on both H3K4 methylation-dependent and -independent mechanisms [78,97,102]. Further molecular dissection of the role of Set1 at telomeres is expected to reveal mechanisms by which it regulates the telomere–chromatin structure and function in yeast and may shed light on the role of H3K4 methylation and Set1 orthologs at mammalian telomeres. Additionally, the methyltransferase Set5, which targets H4K5, K8, and K12 for methylation [103], also appears to play a minor role in regulating telomere gene expression and potentially telomere function, particularly in collaboration with Set1 [78,104]; however, the precise mechanism by which this enzyme acts at telomeres remains to be elucidated in yeast [105], and has not yet been investigated in mammalian systems.

#### 2.1.3. Histone Ubiquitination at Telomeres

The ubiquitination of H2BK123 in budding yeast, mediated by Rad6 and Bre1, is often a prerequisite for the establishment of other histone PTMs, including H3K4me and H3K79me [106,107,108,109]. It is also reported to have a role in replicative lifespan regulation through interaction with the Sir2 pathway [110]. In addition to maintaining the euchromatin–heterochromatin boundary, H2BK123ub has been shown to regulate telomere replication by promoting end-resectioning [111]. Furthermore, when telomeres become uncapped, H2BK123ub restricts the accumulation of single-stranded DNA at telomere ends by the exonuclease Exo1 [112]. The deubiquitination of H2BK123 is performed by the deubiquitinating module of the Spt-Ada-Gcn5 acetyltransferase complex, or SAGA complex, of which one component is Sus1. The loss of Sus1 and the subsequent increase in H2BK123ub has been reported to promote telomere lengthening [113], indicating that the overall balance of this mark supports telomere homeostasis. In humans, the ubiquitination at lysine 120 of H2B is equivalent to H2BK123ub in yeast. While H2BK120ub similarly serves as a prerequisite for the addition of histone H3 methylation marks [114,115], it is not yet clear whether it has additional telomere-end protection roles. Based on the observations from yeast, further investigation of the H2BK120ub in mammalian systems may reveal its contribution to telomere maintenance.

## 3. Chromatin Regulation at Telomeres and Links to Disease

Telomere syndromes, or telomeropathies, are genetic disorders that have generally been linked to germline mutations in genes encoding telomere maintenance factors, including telomerase subunits and the proteins required for its function, such as *DKC1*, as well as telomere protection factors, including shelterin components like *POT1* [116]. These syndromes, including dyskeratosis congenita (DC) and aplastic anemia, are associated with premature telomere shortening. Clinical manifestations of these disorders are thought to primarily arise due to defects in tissues that rely on the high proliferative capacity of their cells and on the maintenance of tissue stem cells; loss of telomerase function during development disproportionately affects these tissues and limits the regenerative capacity of their stem cells [117,118]. While a number of the factors implicated in these syndromes rely on proper chromatin structure at telomeres, the potential for interaction between disease-associated mutations and chromatin-modifying proteins has not been fully investigated. In particular, given that histone modifications in telomeric regions have been shown to change with age [6,7], there may be synergistic interactions between chromatin-regulatory proteins and telomere maintenance factors that warrant further study, particularly during aging.

The pleiotropic nature of mutations in or the dysregulation of chromatin-modifying enzymes make it challenging to attribute cellular abnormalities to defects in the localized function of a chromatin modifier, such as at telomeres. However, some modifiers, such as SIRT6, distinctly manifest phenotypes associated with telomere dysfunction and have molecular roles at telomeres [51,54,55], indicating that they may be critical to the development of telomere-linked pathologies. Future research into human cells focused on further characterizing chromatin modifiers linked to telomere function in model systems will likely identify additional chromatin factors that specifically act to maintain telomere integrity and prevent aging- and senescence-associated diseases.

Intriguingly, there are key examples indicating that the regulation of gene expression via TPE is critical for preventing diseases associated with telomere maintenance defects. Facioscapulohumeral muscular dystrophy, or FSHD, is a myopathy characterized by facial, scapular, and arm muscle atrophy and shows a late onset associated with telomere attrition [119]. The etiology of FSHD has been linked to the upregulation of the gene *DUX4* in a subset of patients, particularly in the presence of short telomeres [120]. In another report, the genes *SORBS2* and *TMEM8C*, each important for muscle differentiation, were also identified as being subject to regulation via TPE in a manner dependent on telomere length and as possibly contributing to the development of FSHD [37]. Together, these data suggest that FSHD may stem from altered expression levels of key genes, which are normally repressed through TPE, which is maintained at healthy telomere lengths but degrades as cells age and telomeres shorten. The precise role of specific histone modifiers and chromatin regulators in the aberrant TPE observed in cells from FSHD patients is not yet clear. Further investigation into the chromatin dynamics and modifications underlying TPE in human cells will not only provide fundamental insight into the factors required for the maintenance of chromatin near telomeres, it may also suggest possible avenues for therapeutic intervention into FSHD and other TPE-linked diseases.

## 4. Conclusions

The examples of chromatin regulation discussed here suggest that the composition and distribution of histone modifications within telomeric regions is important to telomere function, as aberrant patterns of modifications are linked to telomeric instability and altered gene expression patterns in subtelomeres. Studies from model systems, in particular budding and fission yeast, have provided great insight into the role of histone modification marks and chromatin structure in telomere maintenance pathways. Much of the foundational knowledge gained from these systems is also applicable to humans; however, there are still substantial gaps in our understanding of the interplay between chromatin structure, gene repression, and telomere integrity in human cells. Detailed mechanistic studies are required to further elucidate the specific contributions of histone modifications to chromatin regulation near telomeres and their roles in telomere maintenance in mammalian systems. For example, the findings in yeast implicating the conserved marks H2BK123ub and H3K4me in telomere maintenance suggest that the exploration of the roles of these marks in human cells at telomeres may uncover new mechanisms by which telomeric and subtelomeric chromatin are regulated. Furthermore, while studies of some chromatin modifiers, such as SIRT6, and the characterization of some telomere-linked diseases, such as FSHD, indicate a key role for chromatin organization and histone modifications in preventing the accumulation of telomere-linked defects, there are relatively few defined links between chromatin regulation and pathologies associated with abnormal telomere function. Determining the precise contribution of chromatin-modifying proteins to genetic telomeropathies or the epigenetic mechanisms of disease associated with telomere dysfunction, including pathologies associated with aging, will shed light on both basic cellular mechanisms of telomere regulation and genome stability, and open new avenues for alleviating diseases linked to dysfunctional telomeres.
